# High SARS-CoV-2 Exposure in Feline Residents of a Cat Café in Texas, United States, 2021–2022

**DOI:** 10.3390/vetsci12040389

**Published:** 2025-04-21

**Authors:** Cassandra Durden, Lisa D. Auckland, Wendy Tang, Gabriel L. Hamer, Sarah A. Hamer

**Affiliations:** 1 Department of Veterinary Integrative Biosciences, Texas A&M University, College Station, TX 77843, USA; durden.cassandra.j@gmail.com (C.D.); lauckland@cvm.tamu.edu (L.D.A.); 2 Department of Entomology, Texas A&M University, College Station, TX 77843, USA; wendy.tang@ag.tamu.edu (W.T.); ghamer@tamu.edu (G.L.H.)

**Keywords:** feline, SARS-CoV-2, sentinel

## Abstract

Congregate animal settings can serve as foci for the increased transmission of pathogens, including zoonoses. Domestic cats have been shown to be reservoirs for SARS-CoV-2 but the public health importance of infected cats has not yet been determined. A population of indoor-only residential cats at a cat café in central Texas with a high level of human interaction was evaluated for infection with SARS-CoV-2 in a longitudinal study in 2021–2022. Among 25 cats, none were qRT-PCR-positive, while 50% harbored SARS-CoV-2-neutralizing antibodies, including 1 that remained seropositive for >8 months. The high level of human exposure in this unique congregate cat setting—in which dozens of new visitors interact with the cats every day—likely facilitated the human-to-cat transmission of SARS-CoV-2 that led to a 50% infection prevalence in cats. This work was conducted when the Delta and Omicron variants predominated. Given that feline susceptibility to infection and shedding of a virus may vary across different viral variants, veterinary surveillance may be an important component of veterinary and human health risk assessments.

## Simple Summary

Animal infections with the pandemic virus SARS-CoV-2 raise concerns for impacts on animal health and spillover transmission to humans. Different viral variants may impact animals in different ways, so ongoing animal surveillance is critical for veterinary and public health. We tracked a cohort of cats that resided in a cat café, where dozens of patrons visit daily to interact with cats, as this congregate animal setting may represent a setting of high transmission risk due to frequent human–cat interactions. We found that half the cats were exposed and harbored neutralizing antibodies to the virus, demonstrating that such settings may be important in the epidemiology of SARS-CoV-2.

## 1. Introduction

Cat cafés are a popular type of coffee or tea shop which allows customers to play with cats that roam freely around the café. The prominent level of cat–cat and human–cat interactions, including the influx of daily customers, may facilitate the transmission of veterinary and zoonotic pathogens. A recent study found that the average number of times cats in a cat café were sick was greater than that for cats in foster care in the same geographic area [1]. There is evidence of human cases of giardiasis from the zoonotic agent *Giardia duodenalis* originating in cat cafés [2]. Similarly, in China, cat cafés are suspected of contributing to the rise in *Pasteurella multocida* cases in humans [3].

Since its initial discovery in China in 2019, SARS-CoV-2 has been the cause of one of the largest pandemics in human history. SARS-CoV-2 has been confirmed to infect a wide variety of mammalian hosts, which is attributed to its entry via the ACE-2 receptor [4]. Domestic cats may become infected with SARS-CoV-2 through contact with other infected cats, infected humans, or SARS-CoV-2-contaminated environments [5]. While many infected cats are asymptomatic [6], some may display clinical signs similar to those of human COVID-19 infections, including respiratory distress, coughing, sneezing, fever, nasal discharge, and others [7].

In rare circumstances, SARS-CoV-2-infected animals have been the source of infection to humans, including an instance of cat to human transmission [8]. Among the central Texas pets living in houses with confirmed human cases of SARS-CoV-2 early on in the pandemic, 36–43.8% of felines were found to have been infected with the virus [9]. Outside of the home, shelters and farms have also investigated SARS-CoV-2 transmission among felines. In shelters, where cats are often in close and sustained proximity with each other as well as human caregivers, seropositivity ranges from 0.8 to 10% [10,11,12,13]. Cats residing on mink farms have displayed seropositivity as high as 18% [14]. To date, no studies have investigated SARS-CoV-2 transmission dynamics among felines residents in a cat café. In rare circumstances, SARS-CoV-2-infected animals have been the source of infection to humans, including an instance of cat-to-human transmission [8].

## 2. Materials and Methods

We quantified the level of SARS-CoV-2 exposure and infection among the feline residents of a new cat café in Brazos County, Texas, which opened in September 2021. The establishment consisted of an approximate 200 ft^2^ public room with tables, chairs, cat beds, cat trees, and numerous toys and enrichment activities for the cats. To enter the café, customers paid a small fee of USD 7 per hour. The café was open 8 h daily, 7 days per week, and a range of 40–100 unique customers visited the café daily during the period of our study (café management, personal communication). At the café’s opening, all resident cats were purebred and arrived at the café after being purchased from breeders or other homes. While many of the resident cats from the initial sampling point remained at the café through the last sampling point, several others were added through partnerships with local humane societies and adoption from other homes.

Given the total population size of ~30 cats at the cat café, our representation of 25 of these cats across our study affords us 95% confidence that our calculated period prevalence is within 5% of true prevalence, given an anticipated 8% SARS-CoV-2 seroprevalence, based on the literature [15]. Cats were sampled opportunistically at 4 time periods in 2021–2022, approved by TAMU’s Institutional Animal Care and Use Committee. All cats who were approved by the café owner were sampled, with no restrictions in the selection criteria. Information on the sex and breed of cats was included, but information on age was not available. Nasal, oral, rectal, and external body (fur) swabs, immersed into 3 mL viral transport media (VTM; made following CDC SOP#: DSR-052-02), and blood samples were collected.

Swabs were tested for SARS-CoV-2 RNA using qRT-PCR by targeting a conserved region of the RdRp gene. Primers and a probe were designed based on a stable region of the viral genome to ensure broad detection across SARS-CoV-2 variants. The primer sequences used were RdRp_SARSr-F2 (GTGARATGGTCATGTGTGGCGG; 600 nM final concentration, IDT Cat. #10006882) and RdRp_SARSr-R1 (CARATGTTAAASACACTATTAGCATA; 800 nM, IDT Cat. #10006883), with probe sequence RdRp_SARSr-P2 (FAM-CAGGTGGAACCTCATCAGGAGATGC-BBQ; 100 nM, IDT Cat. #10006886). Reactions were carried out using the TaqMan Fast Virus 1-Step Kit (Thermo Fisher Scientific, Waltham, MA, USA) according to the manufacturer’s protocol. Thermal cycling conditions were as follows: reverse transcription at 50 °C for 30 min, initial denaturation at 95 °C for 15 min, followed by 45 amplification cycles of 95 °C for 15 s and 58 °C for 60 s. A synthetic 2019-nCoV_RdRp (ORF1ab) RNA control was included as a positive control in each run.

Sera were assayed for SARS-CoV-2-neutralizing antibodies using plaque reduction neutralization tests against SARS-CoV-2 Isolate USAIL1/2020, NR 52381 (BEI Resources, Manassas, VA, USA) following methods we previously reported [16]. Samples which neutralized viral plaques by 50% or more (PRNT_50_) were interpreted as seropositive. Those that were able to neutralize viral plaques by 90% or more (PRNT_90_) were further tested in 2-fold dilutions, starting at 1:10, to determine 90% endpoint titers. Although the PRNT approach does not afford differentiation between IgG and IgM responses, the presence of neutralizing activity, particularly in the later stages post-infection, is likely attributable to IgG, which is typically responsible for longer-term antiviral immunity.

## 3. Results

In total, 25 unique cats were sampled across four café visits between 30 September 2021, and 14 October 2022, yielding 120 swabs and 40 blood samples in total. All qRT-PCR tests on swabs were negative. Of these 25 cats, 22 were blood-sampled at least once, and 11 of them harbored SARS-CoV-2-neutralizing antibodies using a PRNT_50_ cutoff for a 13-month period prevalence of 50%. Four cats also met the PRNT_90_ positivity criteria, all of which had endpoint titers of 10 (Table 1).

Of these 11 seropositive cats, 3 were male, 8 were female, and 9 were purebred (Table 1). A chi-squared test revealed no significant effect of sex on seropositivity (*p* = 0.0734, Table 2). Additionally, we performed Fisher’s exact test to investigate the effect of breed on seropositivity, and found that purebred cats were significantly more likely to be positive than domestic short- and long-hair cats (*p* = 0.04638, Table 2).

**Figure 1:**
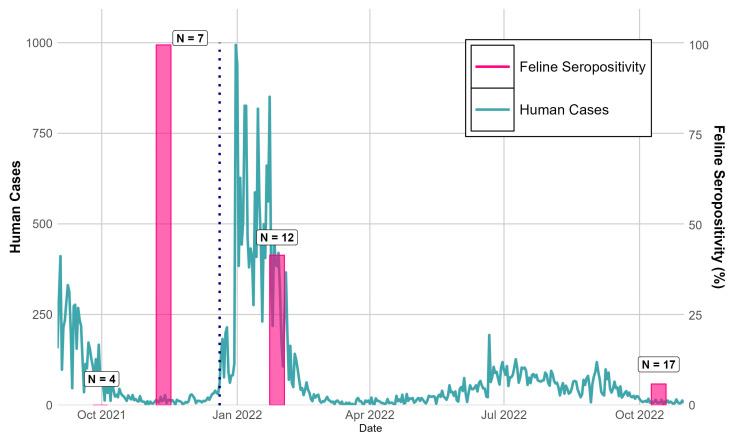
Confirmed new human cases of SARS-CoV-2 in Brazos County, TX, 1 September 2021 to 31 October 2022 vs. proportion of infected cats at a cat café over the study period. Human case data collected from Texas Department of State Health Services (https://www.dshs.texas.gov/covid-19-coronavirus-disease-2019/texas-covid-19-data, accessed on 16 April 2025). The vertical dotted line represents the introduction of the Omicron variant to Brazos County (20 December 2021).

The proportion of cats who were seropositive varied over time, with a peak of 100% (seven out of seven) in November 2021 when Delta was predominant in humans, with a lower proportion of them being seropositive in 2022 once Omicron emerged (Figure 1). Three cats negative on the initial sampling date, 30 September 2021—the same month the café opened to the public—were later positive in November 2021, suggesting new infections were acquired between 30 September and early November 2021, when cats likely became accustomed to frequent interactions with customers. On 28 January 2022, 5 of 12 (41.6%) cats were seropositive. The collection took place after the introduction of Omicron to Brazos County, but Delta was still the primary variant circulating at this time (Figure 1). Four of these twelve cats had been previously sampled in November, including three previously seropositive cats that seroreverted to negative, and one cat that remained positive 2.5 months later. Finally, on 14 October 2022 when the Omicron variant was dominant in the human population, only 1 of the 17 cats tested was seropositive (5.9%), and 7 cats were new to the café and had not been previously sampled. The single seropositive cat was also positive in January 2022, suggesting the retention of neutralizing antibodies for at least 8 months or the acquisition of a new infection, whereas four previously seropositive cats had reverted to seronegative. Consistent with our findings, a prior study showed long-term immunity in domestic cats 3–8 months past the initial positive testing date [17], and immunity to SARS-CoV-2 in cats has been shown to protect against re-infection [13]. Further, the lower proportion of seropositive cats at the end of our study supports the findings of experiments showing that Omicron is less infectious to felines [18]. Notably, our study also did not report the specific variants observed in the cat population of the café, and confirmation of the circulating variant is needed to make strong conclusions about the effect of SARS-CoV-2 variants on feline seropositivity.

## 4. Discussion

Studies of pet cats in households with confirmed COVID-19 cases in Texas, Washington, Utah, Idaho, and Ontario, Canada, showed that 31–52% of cats were seropositive for SARS-CoV-2, with risk factors for cat infection including sleeping in a bed with owners, or being held, petted, and kissed by infected owners [19,20,21]. In contrast, despite the considerable number of human interactions each café cat in our study may have had, the interactions were brief, with most patrons spending only an hour or two at the café, and customers were not allowed to pick up or kiss the cats. This is consistent with research on best practices for human–cat interactions with café cats, which suggest that short, limited touch is ideal for feline comfort in this environment and may have mitigated further SARS-CoV-2 transmission. Additional protective behaviors, including visitor screening and the isolation of sick cats, could also be employed at cat cafés and similar environments.

We found that the highest seropositivity throughout the study was in January of 2021, when the Delta variant was prominent in the human population throughout Brazos County. Conversely, we found the lowest seropositivity after the Omicron variant became predominant. Similarly, other studies have found higher rates of feline infection with the Delta variant compared to the Omicron variant, both in laboratory and field settings, and our findings may support this idea [18,22,23].

Purebred cats may have increased susceptibility to several genetic and infectious diseases due to the nature of their breeding [24,25]. Many of the resident cats in the café were purebred (68%). We found that purebred cats were significantly more likely to have SARS-CoV-2-neutralizing antibodies compared to non-purebred cats. This is consistent with previous studies which found domestic purebred and pedigree cats more susceptible to the SARS-CoV-2 than non-purebred cats, potentially owing to the genetics associated with selective breeding [26].

Sampling bias may have been introduced into the study design because not all cats were made available by the café owner at all sampling time points, with a couple of cats being restricted from the research team due to owner-disclosed behavioral problems or sickness. However, their omission from the study does not change the infection outcomes in the cats we measured. Further, given our small sample size, limited sampling periods, and the fact that we only sampled one café, our study may not be representative of the risk of feline SARS-CoV-2 infection across all cat cafés or aggregate cat settings. This café in Brazos County ultimately closed in February of 2023, citing financial issues as a reason. Notably, the café posted that they were dealing with several cases of zoonotic pathogens in the café during the period of sampling on their social media, including cases of *Tritrichomonas foetus*, ringworm (*Microsporum canis*), and *Bartonella.* Zoonotic disease mitigation should be considered in all cat cafés.

## 5. Conclusions

Cat cafés may be high-risk settings for the transmission of SARS-CoV-2, with varying patterns of cat infection across different waves of the pandemic. Such residential cats with no or limited travel outside of the café may serve as effective sentinels for the dynamics of transmission in the local human community.

## Figures and Tables

**Table 1:** Cats sampled for SARS-CoV-2-neutralizing antibodies at a cat café over 13 months (September 2021–October 2022), Texas, USA. Seropositivity is denoted for positive samples capable of neutralizing 50 and 90% of viral plaques (PRNT 50 and PRNT 90, respectively). NEG indicates a cat was not positive at that date, while NA represents cats not sampled on a given date. Seropositive cats are listed first (rows 1–11), followed by seronegative cats (12–25).

Feline ID	Breed	Sex	30 September 2021	12 November 2021	28 January 2022	14 October 2022
1	Maine coon	M	NEG	NA	POS (PRNT_50_)	NEG
2	Scottish Fold	F	NEG	POS (PRNT_50_)	NEG	NEG
3	American Short Hair	M	NEG	POS (PRNT_90_)	NEG	NEG
4	Turkish Angora	F	NEG	POS (PRNT_90_)	NEG	NEG
5	American Short Hair	F	NA	NA	POS (PRNT_90_)	POS (PRNT_50_)
6	Maine coon	F	NA	NA	POS (PRNT_50_)	NEG
7	Turkish Angora	F	NA	POS (PRNT_90_)	POS (PRNT_50_)	NEG
8	Ragdoll	F	NA	POS (PRNT_50_)	NEG	NA
9	Siamese	M	NA	POS (PRNT_50_)	NEG	NA
10	Domestic Short Hair	F	NA	POS (PRNT_50_)	NA	NA
11	Bengal	F	NA	NA	POS (PRNT_50_)	NA
12	Siamese	M	NA	NA	NEG	NEG
13	Ragdoll	M	NA	NA	NA	NEG
14	Munchkin	M	NA	NA	NA	NA
15	Persian + Munchkin	M	NA	NA	NA	NEG
16	Domestic Short Hair	F	NA	NA	NA	NEG
17	Persian	M	NA	NA	NA	NEG
18	Domestic Short Hair	M	NA	NA	NA	NEG
19	Bengal	M	NA	NA	NA	NEG
20	Munchkin	F	NA	NA	NA	NEG
21	Domestic Long Hair	M	NA	NA	NA	NEG
22	British Short Hair	F	NA	NA	NA	NEG
23	Domestic Short Hair	M	NA	NA	NA	NEG
24	Domestic Short Hair	F	NA	NA	NA	NEG
25	Unknown	M	NA	NA	NA	NEG

**Table 2:** Cats tested for SARS-CoV-2-neutralizing antibodies by sex and breed. Positives include all seropositivity across the study period. Cats in the “Other” category include domestic short- and long-hair cats.

Breed	Positive	Negative
Other	0	5
Purebred	11	6
95% CI: 1.157—Inf	OR: INF	* *p*-value: 0.0351
Sex		
Male	3	8
Female	8	3
X-squared = 2.9091	df = 1	*p*-value: 0.0881

* indicates significance at α = 0.05.

## Data Availability

The original contributions presented in this study are included in the article. Further inquiries can be directed to the corresponding author(s).

## Abbreviations

The following abbreviations are used in this manuscript:

ACE-2	Angiotensin-converting enzyme 2
COVID-19	Coronavirus disease 2019
PRNT	Plaque reduction neutralization test
SARS-CoV-2	Severe acute respiratory syndrome coronavirus 2
qRT-PCR	Quantitative reverse transcriptase polymerase chain reaction
VTM	Viral transport media
